# Coverage of the requirements of first and second level stroke unit in Italy

**DOI:** 10.1007/s10072-020-04616-x

**Published:** 2020-07-31

**Authors:** Monica Bandettini di Poggio, Danilo Toni, Carlo Gandolfo, Damiano Paolicelli, Andrea Zini, Elio Agostoni, Fabio Bandini, Michele Ragno, Maria Concetta Altavista, Antonio Bertolotto, Gabriele Siciliano, Michele Vecchio, Nicola Tambasco, Antonio Gambardella, Paolo Manganotti, Maurizio Melis, Marco Onofrj, Giuseppe De Michele, Nicoletta Reale, Gioacchino Tedeschi, GianLuigi Mancardi

**Affiliations:** 1 Department of Neuroscience, Rehabilitation, Ophthalmology, Genetics, Maternal and Child Health (DINOGMI), Policlinic San Martino Hospital, University of Genova, Genoa, Italy; 2 Neurovascular Unit, Policlinico Umberto I, Department of Neurology and Psychiatry, University of Rome, ‘La Sapienza’, Rome, Italy; 3 Dipartimento di Scienze Mediche di Base, Neuroscienze ed Organi di Senso, Università degli Studi di Bari “A. Moro”, Bari, Italy; 4 IRCCS Istituto di Scienze Neurologiche di Bologna, Maggiore Hospital, Bologna, Italy; 5 Department of Neurology, Niguarda Ca’ Granda Hospital, Milan, Italy; 6 Department of Neurology, S.Paolo Hospital, Savona, Italy; 7 Neurologia AV5, Ascoli Piceno-, San Benedetto del Tronto, Italy; 8 UOC di Neurologia, ASL Roma 1, Presidio Ospedaliero San Filippo Neri, Rome, Italy; 9 SCDO Neurologia & CRESM, AOU San Luigi, Orbassano, Italy; 10 Dipartimento di Medicina Clinica e Sperimentale, Clinica Neurologica, Università di Pisa e Azienda Ospedaliero Universitaria Pisana, Pisa, Italy; 11 U.O. of Physical Medicine and Rehabilitation, Policlinico Vittorio Emanuele University Hospital, University of Catania, Catania, Italy; 12 Clinica Neurologica, Azienda Ospedaliera e Universitaria di Perugia, Perugia, Italy; 13 Medical and Surgical Sciences Department, School of Medicine, Magna Græcia University of Catanzaro, Viale Europa, Catanzaro, Italy; 14 Clinical Unit of Neurology, Department of Medicine, Surgery and Health Sciences, Cattinara University Hospital ASUGI, University of Trieste, Trieste, Italy; 15 SC Neurologia e Stroke Unit, Azienda Ospedaliera G.Brotzu, Cagliari, Italy; 16 Clinica Neurologica, Dipartimento di Neuroscienze, Imaging e Scienze Cliniche, Università Chieti-Pescara, Chieti, Italy; 17 Dipartimento di Neuroscienze e Scienze Riproduttive ed Odontostomatologiche, Università di Napoli Federico II, Naples, Italy; 18 Association for the Fight against Stroke (A.L.I.Ce.) Italia Odv, Rome, Italy; 19 Department of Advanced Medical and Surgical Sciences, University of Campania “Luigi Vanvitelli”, Naples, Italy; 20 Department of Neuroscience, Rehabilitation, Ophthalmology, Genetics, Maternal and Child Health, University of Genova and IRCCS ICS Maugeri, Genova, Pavia, Italy

**Keywords:** Stroke, Stroke unit, Neurointerventionists, Thrombolysis

## Abstract

**Background and aim:**

In the scientific literature, there is unanimous consensus that hospitalization in stroke unit (SU) is the most important treatment for stroke patients. In this regard, the Act number 70/2015 by the Italian government identified specific skills that contribute to a classification of SU and outlined a “hub and spoke” stroke network. The aim of our study was to check the coverage of requirements of first and second level SU in the national territory and to shed light on any deficit or misdistribution of resources.

**Material and methods:**

In 2019, a survey on the current situation related to stroke care in Italy was carried out by the Italian Society of Neurology (SIN), The Italian Stroke Organization (ISO), and the Association for the Fight against Stroke (A.L.I.Ce).

**Results:**

First level SU was found to be 58 against a requirement, according to the Act 70/2015, of 240. Second level SU was found to be 52 compared with an expected requirement of 60. Neurointerventionists were 280 nationally, with a requirement of 240. A misdistribution of resources within individual regions was often seen.

**Conclusions:**

The survey demonstrated a severe shortage of beds dedicated to cerebrovascular diseases, mainly because of lack of first level SU, especially in central and southern Italy. It also suggests that the current hub and spoke system is not yet fully implemented across the country and that resources should be better distributed in order to ensure uniform and fair care for all stroke patients on the whole territory.

**Electronic supplementary material:**

The online version of this article (10.1007/s10072-020-04616-x) contains supplementary material, which is available to authorized users.

## Introduction

In the scientific literature, there is unanimous consensus that hospitalization in stroke unit (SU) is the most important treatment for the generality of stroke patients. The benefit in terms of better long-term outcome of acute stroke patients admitted into SU versus conventional wards has been previously demonstrated in several randomized trials and their meta-analysis [1]. Two Italian observational follow-up studies confirmed SU patient management as predictor of good outcome in a real-world setting, across all age ranges and clinical characteristics [2, 3]. In this regard, both European and Italian guidelines suggest transporting all cases of suspected stroke to the emergency room of the nearest hospital provided with SU [4, 5]. The Act number 70/2015 by the Italian government identified specific skills that contribute to a classification of stroke unit (Table 1). First level SU are characterized by the presence of at least one dedicated neurologist, a committed nursing staff, at least one bed with continuous monitoring, and the possibility of carrying out intravenous thrombolytic therapy (IVT) and connection with a 2nd level stroke center. Second level SU must treat at least 500 stroke cases/year, have dedicated staff 24 h a day, have neuroradiology active 24/7, and be able to perform mechanical thrombectomy in emergency (Table 1). The evidence is that stroke care involves management by a dedicated stroke team consisting of vascular neurologist, neurosurgeon, neurointerventionist, radiologist, anesthesiologist, specialized nurses, other trained medical personnel, and rehabilitation facilities. It also requires the availability of cutting-edge technology at their disposal. Since this requires high costs and it is impossible to have all these facilities in every hospital in a given area, the government Act 70/2015 identified a “hub and spoke” stroke network. According to this model, a single hospital staffed by specialized physicians and with the appropriate high-technology infrastructure forms the hub (second level emergency departments—EDs), while other hospitals providing less complex forms of care act as the spoke (first level EDs). The Act number 70/2015 states also that a first level SU must be located in a first level ED, with a catchment area of ​​between 150,000 and 300,000 inhabitants, and a second level SU must be located in the second level EDs, with a catchment area of ​​between 600,000 and 1,200,000 inhabitants. Made these premises, the need in Italy for first level stroke centers is 240 and for second level stroke centers is 60, which should be adequately and rationally distributed throughout the national territory. Referring to the “Notebooks of the Ministry of Health on the Organization of Stroke Assistance: The Stroke Units” n.2 of March–April 2010, the need for dedicated beds is about 8 beds in SU every 150–300,000 inhabitants, corresponding to about 1 bed every 19,000 inhabitants.

**Table 1:** Classification of stroke unit as defined by the current Act 70/2015 of the Italian health government

First level stroke unit	Multiprofessional competences into hospital
	Dedicated neurologist and nurses
	One bed with continuous monitoring, at least
	Early rehabilitation
	Intravenous thrombolysis
	Neurosurgical on-call availability (also in other hospital)
	Neurosonology with Doppler ultrasound and echocardiography 24/7 availability of CT scan and CT angiography (at least 16 multislices) and/or MR (also with DWI) and MR angiography
	Linking with second level stroke units and rehabilitation units
Second level stroke unit	The same as first level and:
	500 admissions/year at least
	24/4 neuroradiology with CT (64 multislices) and CT angiography, MR with DW and PW images and MR angiography
	Endovascular interventional unit
	24/7 neurosurgery
	24/7 vascular surgery
	Cerebral angiography
	Intra-arterial thrombolysis (urgency), mechanical thrombectomy (urgency), extra- and intracranial stenting
	Urgent embolization of arteriovenous malformations and aneurisms, endarterectomy
	Decompressive hemicraniectomy
	Aneurismal clipping

## Aim

The aim of this study was to check the coverage of the requirements of first and second level SU in the national territory and to shed light on any deficit or misdistribution of resources.

## Material and methods

In 2019, the Italian Society of Neurology (SIN), in collaboration with A.L.I.Ce. (Association for the Fight against Stroke) and ISO (The Italian Stroke Organization), carried out a study on the current situation related to stroke care in Italy, region by region. Detailed information was collected by regional secretaries of SIN.

The estimate of possible deficiencies was based on the terms of the Act 70/2015, even if for this census, we used a more permissive method, knowing in advance that the healthcare situation in our country was not generally totally overlapping with ministerial indications [6]. We therefore decided that minimum requirements to meet the criterion for first level SU should be (a) the presence of at least one dedicated neurologist or (not “and”) specialized nurses; (b) at least one bed with continuous monitoring; and (c) capability of performing intravenous thrombolysis (IVT) 24/7. On the other hand, we considered second level SU all those centers able to perform mechanical thrombectomy (MT) 24/7 with a team of neurointerventionists of at least 4 units, even if other criteria set out in the Act 70/2015 were not completely met.

Using this method, we separately analyzed (a) the number of first level and second level SU; (b) the number of beds in SU (beSU); (c) the number of stroke beds in traditional wards such as EDs or neurological departments (beTW); (d) the total number of beds dedicated to stroke (TObe); and (e) the number of neurointerventionists able to perform mechanical thrombectomies.

To obtain this information, we created a CRF to be sent to all regional SIN secretaries who were asked to fill in the requested information fields, city by city. Data were then delivered to the administrative office of SIN and were carefully reviewed by authors of this study.

## Results

First level SU (as defined by our “permissive” method) was found to be 58 against a requirement, according to the Act 70/2015, of 240. Second level SU (as defined by our “permissive” method) was found to be 52 compared with an expected requirement of 60.

The number of beds available in SU (beSU) was 723, compared with an expected of 1920. When we also considered those beds dedicated to acute cerebrovascular disorders in traditional wards (beTW) outside SU, the total number of beds increased to 1176 (beSU + beTW), always lower than the need as established by the Ministry of Health (1920 beds).

Neurointerventionists (NIs) (in the vast majority neuroradiologists or radiologists) were 280 nationally, with a requirement of 240.

Detailed data, region by region, are available as supplementary material.

**Fig. 1:**
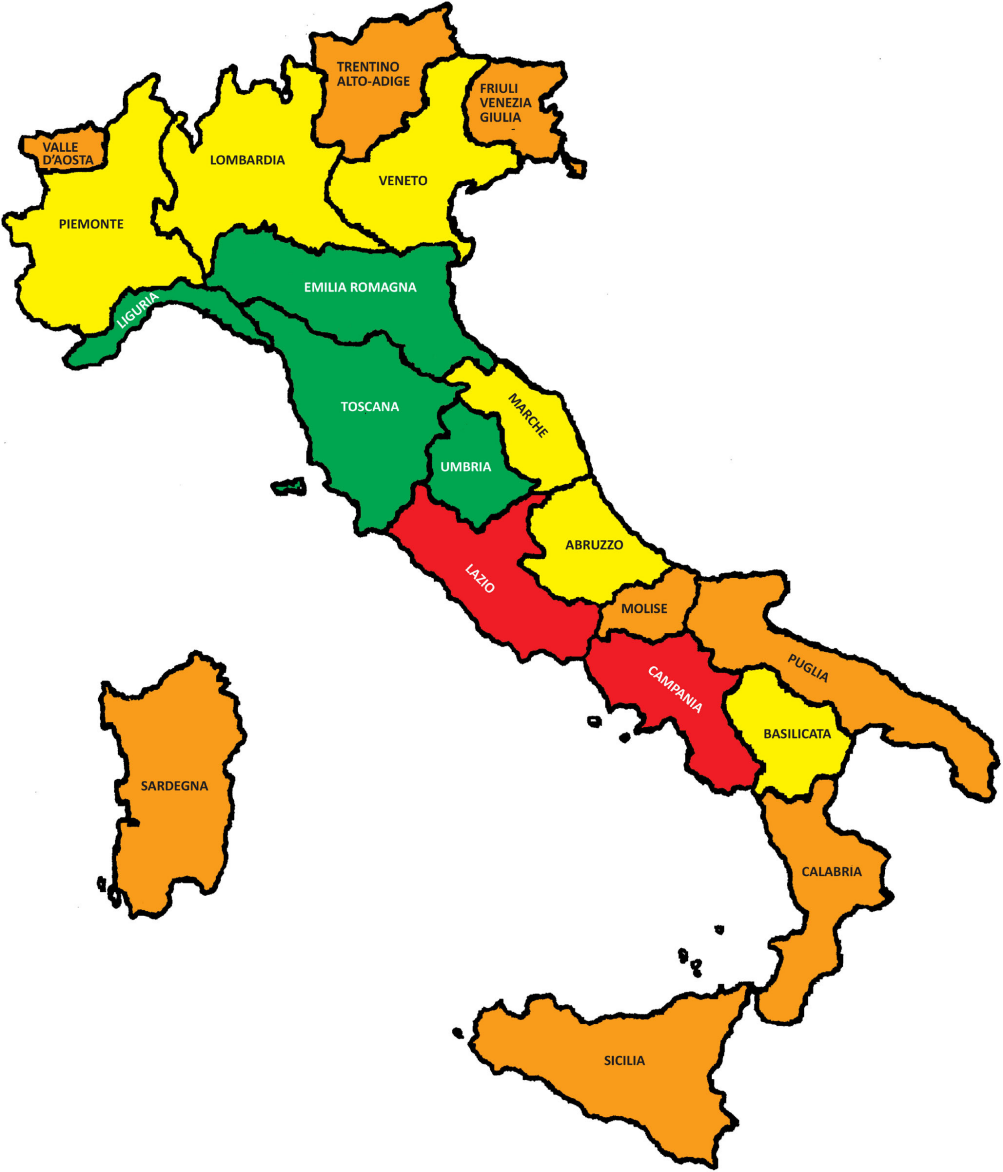
Extent of the lack of beds dedicated to stroke compared with the expected in Italy

**Fig. 2:**
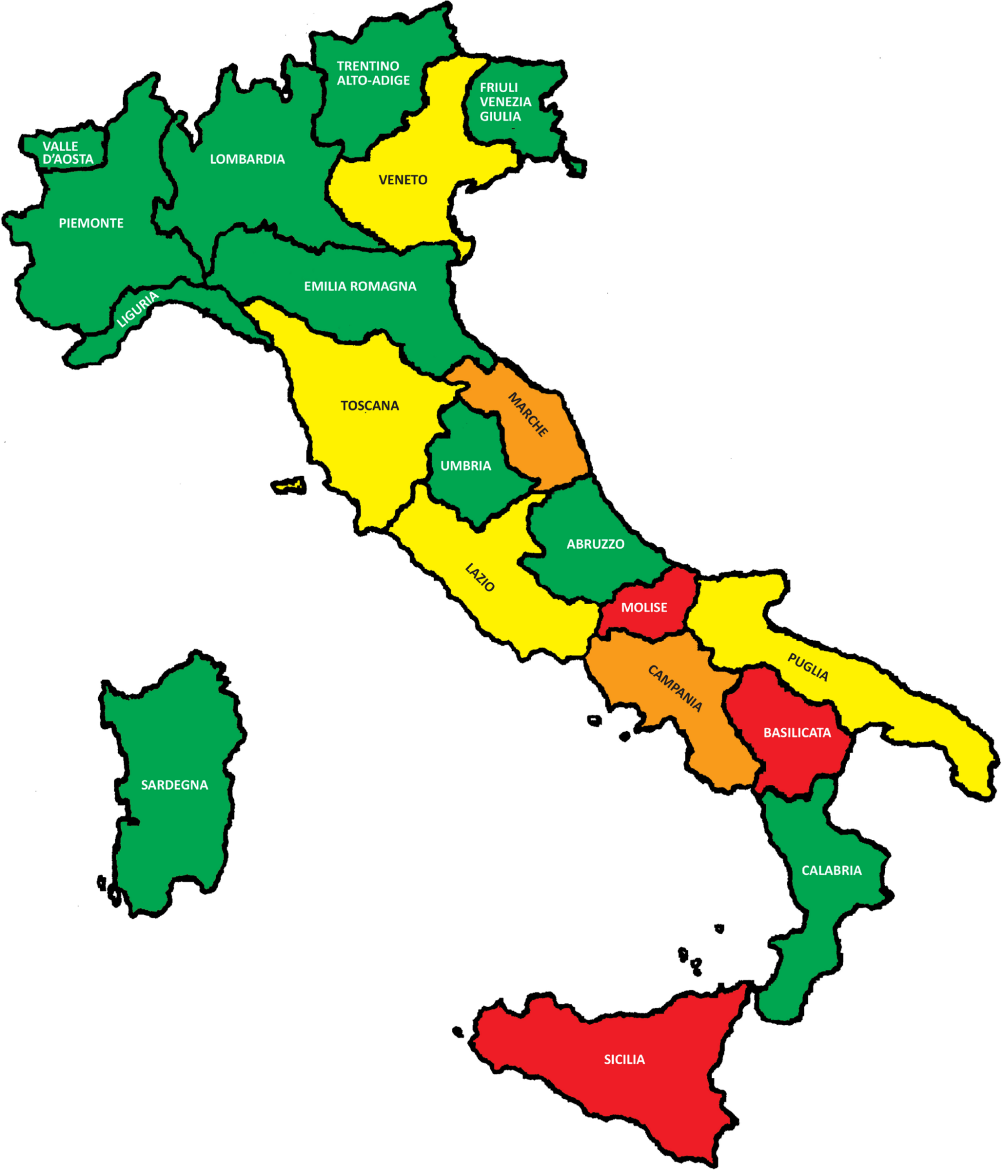
Extent of the lack of neurointerventionists in Italy

In Figs. 1 and 2, we have depicted each region with a different color depending on the degree of shortage of beds dedicated to stroke and of NIs compared with the expected. Red color indicates that the shortage is 51% greater than the requirement, orange between 50 and 31%, yellow between 30 and 11%, and green ≤ 10%. The results indicate a severe shortage of dedicated beds to cerebrovascular patients in central and southern Italy. In addition, we have often seen a misdistribution of resources within individual regions. As it is shown in the supplementary material, significant differences in coverage are not uncommon depending on the provinces. This applies to stroke unit beds and even more to neurointerventionist services. So, regions that appear to be “green” in the map still present problems within them.

## Discussion

This survey shows a severe shortage of beds dedicated to cerebrovascular diseases, mainly because of lack of first level SU. In front of a requirement of about 240 first level SU, our census found only 58 of them, resulting in a shortfall of 76%. More generally when we refer to beds dedicated to cerebrovascular patients, the deficiency is still about 40%. The lack of SU is particularly disadvantageous because of its known benefit in terms of better assistance and long-term outcome.

In fact, if the treatment of choice for ischemic stroke patients is IVT in combination eventually with MT, the shortage of first and second level SU might explain, at least in part, the rate of patients who undergo reperfusion therapies in our country. As proof of this, we compared the rate of IVT in a certain region (data extracted from the Safe Implementation of Treatments in Stroke (SITS) Registry) with the rate of available SU in the same area and noticed a certain correspondence (www.sitsinternational.org). For example, for Liguria and Tuscany, which resulted in our study with a shortage of cerebrovascular beds ≤ 10% than the required, data from the SITS showed a high rate of IVT. By contrast, data from the SITS are less encouraging for regions such as Lazio and Campania, depicted in red on our map in Fig. 1.

However, the beneficial effects of SU admission are not depending only on the higher probability of being treated with reperfusional therapies, but mainly on the patient management in SU independently [2]. As it is known, the benefit comes from the presence of multidisciplinary team with expert vascular neurologists and dedicated nurses, better parameter monitoring in the acute phase and early access to intensive rehabilitation. Unfortunately, in Italy, evidence of SU deficiency has been known for at least 15 years. For example, in 2006, data from a survey carried out by the PROSIT study group showed that in 2003/04, only 9% of the hospital services had organized SU care [6]. Since then, the number of SUs increased thanks to the approval of intravenous thrombolysis only in that setting, and the quality of acute stroke care improved, but the situation has changed only partially and inhomogenously among different regions. In this scenario, we believe that a major health policy maneuver supported by national legislation is required in order to adjust national stroke care to what is required by the ministerial Act 70/2015. In fact, in the era of thrombectomy, it is important that we keep a population perspective in order to appropriately treat most stroke patients who only require medical treatment. In fact, although every patient with stroke is eligible for dedicated stroke unit care, some 25% of the patients will be eligible for IVT, and only an estimated 10% will be eligible for MT [7].

In this desirable healthcare reform, efforts should be made also to increase the availability of neurologist dedicated to stroke (vascular neurologist, VN) in order to break the vicious circle of general neurologists being uncomfortable with managing acute stroke patients because of lack of enough exposure and experience. A VN should be a specialist in neurology with a profound knowledge on stroke causes, its pathogenic mechanisms, and therapeutic possibilities. He should have extensive clinical skills for facing the complex profile of acute stroke patients and not only the manual ability to recanalize the occluded arterial vessel. Even if our study does not have the power to estimate the lack of VNs, recent data have been published on this topic [8], and in agreement with the authors, we believe that progress in the management of acute stroke has the risk of becoming meaningless if there is no workforce able to implement those standards.

On the other hand, our survey found that the number of NIs was higher than expected, theoretically suggesting that endovascular recanalization should be appropriately managed in our country. Unfortunately, the high number of NIs is not synonymous with optimal 24/7 coverage of NIs services. Main potential causes of this partial ineffectiveness of the system are (a) the presence of a number of NI services higher than required in some regions, but with sufficient staff only in some SU (considering a minimum of at least 4 NIs per center to provide the necessary coverage 24/7); (b) misdistribution of services in the regional territory with disparities in coverage depending on the provinces (e.g., in Lazio; see supplementary material); and (c) absence or extreme deficiency of endovascular services in some regions (e.g., in Molise, Basilicata, Sicilia).

This survey suggests that the current hub and spoke system is not yet fully implemented across the country and that NI resources should be better distributed in order to ensure uniform and fair care for all stroke patients on the whole territory. Our study was not designed with the aim to identify the discipline of origin of NIs (radiology, neuroradiology, or neurology) and their average age. Even if our available data show that interventionists mostly adhere to neuroradiology, a better characterization of national NIs should be object of further surveys. Considering the extremely fast growth of interventional neuroradiology and the inevitable generational change that will take place in the next years, a European neuroradiological multi-society group recently set up recommendations for the development of MT services, including consensus on the minimum requirements for centers providing such treatment and training requirements in interventional neuroradiology [9]. Despite differences in resources across Italian healthcare facilities, we believe that efforts should be made to implement these recommendations as a standard of care.

## Electronic supplementary material

ESM 1 (DOCX 19 kb)

ESM 2 (DOCX 22 kb)

ESM 3 (DOCX 28 kb)

ESM 4 (DOCX 19 kb)

ESM 5 (DOCX 21 kb)

ESM 6 (DOCX 23 kb)

ESM 7 (DOCX 41 kb)

ESM 8 (DOCX 20 kb)

ESM 9 (DOCX 18 kb)

ESM 10 (DOCX 27 kb)

ESM 11 (DOCX 22 kb)

ESM 12 (DOCX 24 kb)

ESM 13 (DOCX 36 kb)

ESM 14 (DOCX 30 kb)

ESM 15 (DOCX 53 kb)

ESM 16 (DOCX 24 kb)

ESM 17 (DOCX 15 kb)

ESM 18 (DOCX 25 kb)

ESM 19 (DOCX 37 kb)

ESM 20 (DOCX 28 kb)
