# Supplementary material for: Coverage of the requirements of first and second level stroke unit in Italy
Source: Neurol Sci. 2020 Jul 31;42(3):1073–9. doi: 10.1007/s10072-020-04616-x (PMC7870770; doi:10.1007/s10072-020-04616-x)
Supplement: Supplementary file 1 — (DOCX 19 kb) [file 10072_2020_4616_MOESM1_ESM.docx]

| **Region**  **(3.736.968 ab)** | **Tuscany** | **Tuscany** | **Tuscany** | **Tuscany** | **Tuscany** | **Tuscany** | **Tuscany** | **Tuscany** | **Tuscany** | **Tuscany** |
| --- | --- | --- | --- | --- | --- | --- | --- | --- | --- | --- |
| **City or Town** | pontedera | cecina | piombino | Portoferaio | ATSE M.varchi | AVC FI-SMN | AVC FI-OSMAnnunz  Ponte Nicch | Pistoia | Pescia | Mugello |
| **I level SU** | 0 | 0 | 0 | 0 | 0 | 0 | 0 | 1 | 0 | 0 |
| **II level SU** | 0 | 0 | 0 | 0 | 0 | 0 | 0 | 0 | 0 | 0 |
| **beSU** | 0 | 0 | 0 | 0 | 0 | 0 | 0 | 4 | 0 | 0 |
| **beTW** | 2 | 1 | 1 | 1 | 4 | 4 | 4 | 0 | 4 | 2 |
| **MT 24/7** | no | no | no | no | no | no | no | no | no | no |
| **N. of NIs** | 0 | 0 | 0 | 0 | 0 | 0 | 0 | 0 | 0 | 0 |

| **Region (3.736.968 inhab** | **Tuscany** | **Tuscany** | **Tuscany** | **Tuscany** | **Tuscany** | **Tuscany** | **Tuscany** | **Tuscany** | **Tuscany** | **Tuscany** | **Tuscany** | **Tuscany** | **Total** |
| --- | --- | --- | --- | --- | --- | --- | --- | --- | --- | --- | --- | --- | --- |
| **City or Town** | Pisa | Massa | Lucca | Versilia | Livorno | Siena | Grosseto | Arezzo | Firenze | AVC FI-SGDD | prato | Empoli |  |
| **I level SU** | 0 | 1 | 1 | 0 | 0 | 0 | 0 | 1 | 0 | 0 | 1 | 0 | 5 |
| **II level SU** | 1 | 0 | 0 | 0 | 0 | 1 | O | 0 | 1 | 0 | 0 | 0 | 3 |
| **beSU** | 8 | 5 | 5 | 0 | 0 | 14 | 0 | 10 | 6 | 0 | 8 | 0 | 60 |
| **beTW** | 0 | 0 | 0 | 4 | 4 | 0 | 4 | 0 | 0 | 4 |  | 7 | 46 |
| **MT 24/7** | yes | no | no | no | no | yes | no | no | yes | no | no | no | 3 |
| **N. of NIs** | 3 | 0 | 0 | 0 | 0 | 5 | 0 | 0 | 5 | 0 | 0 | 0 | 13 |

Legend: SU, stroke unit; beSU, beds available in SU; beTW, beds available in traditional wards; MT, Mechanical thrombectomy ; NIs, Neuro interventionists; uk, unknown the number of available beds
