# Supplementary material for: Coverage of the requirements of first and second level stroke unit in Italy
Source: Neurol Sci. 2020 Jul 31;42(3):1073–9. doi: 10.1007/s10072-020-04616-x (PMC7870770; doi:10.1007/s10072-020-04616-x)
Supplement: Supplementary file 2 — (DOCX 22 kb) [file 10072_2020_4616_MOESM2_ESM.docx]

| **Region (**1,062,860 inhab.) | **Trentino-Alto-Adige** | | **Total** |
| --- | --- | --- | --- |
| **City/Town** | Bolzano | Trento |  |
| **I level SU** | 0 | 0 | 0 |
| **II level SU** | 1 | 1 | 2 |
| **beSU** | 8 | 8 | 16 |
| **beTW** | 0 | 0 | 0 |
| **MT24/7** | yes | Yes | 2 |
| **N. of NIs** | 4 | 4* | 4 |

Legend: SU, stroke unit; beSU, beds available in SU; beTW, beds available in traditional wards; MT, Mechanical thrombectomy; NIs, Neuro interventionists; * the operators are shared
