# Supplementary material for: Coverage of the requirements of first and second level stroke unit in Italy
Source: Neurol Sci. 2020 Jul 31;42(3):1073–9. doi: 10.1007/s10072-020-04616-x (PMC7870770; doi:10.1007/s10072-020-04616-x)
Supplement: Supplementary file 5 — (DOCX 21 kb) [file 10072_2020_4616_MOESM5_ESM.docx]

| **Region (4,907,529 inhab.)** | **Veneto** | | | | | |
| --- | --- | --- | --- | --- | --- | --- |
| **City/Town** | Vicenza | Verona | Treviso | Rovigo | Padova | Venezia Mestre |
| **I level SU** | 0 | 0 | 0 | 0 | 0 | 0 |
| **II level SU** | 1 | 1 | 1 | 1 | 1 | 1 |
| **beSU** | 8 | 8 | 8 | 6 | 4 | 5 |
| **beTW** | 0 | 0 | 0 | 0 | 0 | 0 |
| **MT 24/7** | yes | yes | yes | yes ˟ | yes | yes |
| **N. of NIs** | 2 | 4 | 2 | 1 | 4 | 4 |

| **Region** | **Veneto** | | | | | | |
| --- | --- | --- | --- | --- | --- | --- | --- |
| **City/Town** | Bassano del grappa | Arzignano | Thiene | Legnago | Peschiera | Negrar | Mirano |
| **I level SU** | 1 | 1 | 1 | 1 | 1 | 1 | 1 |
| **II level SU** | 0 | 0 | 0 | 0 | 0 | 0 | 0 |
| **beSU** | 4 | 4 | 6 | 4 | 4 | 4 | 4 |
| **beTW** | 0 | 0 | 0 | 0 | 0 | 0 | 0 |
| **MT 24/7** | 0 | 0 | 0 | 0 | 0 | 0 | 0 |
| **N. of NIs** | 0 | 0 | 0 | 0 | 0 | 0 | 0 |

| **Region** | **Veneto** | | | | | | |
| --- | --- | --- | --- | --- | --- | --- | --- |
| **City/Town** | Portogruaro | Venezia | Belluno | Feltre | Castelfranco | Schiavonia | Cittadella |
| **I level SU** | 1 | 1 | 1 | 1 | 1 | 1 | 1 |
| **II level SU** | 0 | 0 | 0 | 0 | 0 | 0 | 0 |
| **beSU** | 2 | 2 | 2 | 2 | 4 | 4 | 4 |
| **beTW** | 0 | 0 | 0 | 0 | 0 | 0 | 0 |
| **MT 24/7** | 0 | 0 | 0 | 0 | 0 | 0 | 0 |
| **N. of NIs** | 0 | 0 | 0 | 0 | 0 | 0 | 0 |

| **Region** | **Veneto** | | | **Total** |
| --- | --- | --- | --- | --- |
| **Citty/Town** | Osp. S. Antonio-Padova | Piove di Sacco | Santorso |  |
| **I level SU** | 1 | 0 | 1 | 17 |
| **II level SU** | 0 | 0 | 0 | 6 |
| **beSU** | 8 | 2 | 2 | 101 |
| **beTW** | 0 | 0 | 0 | 0 |
| **MT 24/7** | 0 | 0 | 0 | 6 |
| **N. of NIs** | 0 | 0 | 0 | 17 |

Legend: SU, stroke unit; beSU, beds available in SU; beTW, beds available in traditional wards; MT, Mechanical thrombectomy; NIs, Neuro interventionists;* the service is active, but not 24/7
