# Supplementary material for: Coverage of the requirements of first and second level stroke unit in Italy
Source: Neurol Sci. 2020 Jul 31;42(3):1073–9. doi: 10.1007/s10072-020-04616-x (PMC7870770; doi:10.1007/s10072-020-04616-x)
Supplement: Supplementary file 6 — (DOCX 23 kb) [file 10072_2020_4616_MOESM6_ESM.docx]

| **Region (1,648,176 inhab.)** | **Sardegna** | | | **Total** |
| --- | --- | --- | --- | --- |
| **City/Town** | Cagliari | Nuoro | Sassari |  |
| **I level SU** | 0 | 0 | 0 | 0 |
| **II level SU** | 1 | 1 | 1 | 3 |
| **beSU** | 13 | 0 | 6 | 19 |
| **beTW** | 0 | 8 | 0 | 8 |
| **MT24/7** | yes | yes | yes | 3 |
| **N. of NIs** | 5 | 3 | 4 | 12 |

Legend: SU, stroke unit; beSU, beds available in SU; beTW, beds available in traditional wards; MT, Mechanical thrombectomy; NIs, Neuro interventionists
