# Supplementary material for: Coverage of the requirements of first and second level stroke unit in Italy
Source: Neurol Sci. 2020 Jul 31;42(3):1073–9. doi: 10.1007/s10072-020-04616-x (PMC7870770; doi:10.1007/s10072-020-04616-x)
Supplement: Supplementary file 7 — (DOCX 41 kb) [file 10072_2020_4616_MOESM7_ESM.docx]

| **Region (4,983,000 inhab.)** | **Sicilia** | | | | | | | |
| --- | --- | --- | --- | --- | --- | --- | --- | --- |
| **City/Town** | Civico -Palermo | Villa Sofia-Cervello-  Palermo | Sant’Antonio Abate-  Trapani | Policlinico P. Giaccone-Palermo | Ospedale Buccheri_La Ferla-  Palermo | Fondaz G. Giglio-Cefalù | AO Cannizzaro-  Catania | PO San Giovanni di Dio-  Agrigento |
| **I level SU** | 0 | 0 | 0 | 0 | 0 | 0 | 1 | 0 |
| **II level SU** | 0 | 0 | 0 | 0 | 0 | 0 | 0 | 0 |
| **beSU** | 0 | 0 | 0 | 0 | 0 | 0 | 6 | 0 |
| **beTW** | 8 | 6 | 4 | 4 | 4 | 4 | 0 | 4 |
| **MT24/7** | no* | 0 | 0 | 0 | 0 | 0 | no* | 0 |
| **N. of NIs** | 1 | 0 | 0 | 0 | 0 | 0 | 1 | 0 |

| **Region** | **Sicilia** | | | | | | | | **Total** |
| --- | --- | --- | --- | --- | --- | --- | --- | --- | --- |
| **City/Town** | ARNAS Garibaldi-  Catania |  | PO Umberto I-Siracusa | PO Guzzardi di Vittoria | AOU Policlinico G. Martino  Messina | S. Agata Militello - PO Generale  Messina | PO Sant’Elia  Caltanisetta | Umberto I (EN) |  |
| **I level SU** | 0 | 0 | 0 | 0 | 0 | 0 | 0 | 0 | 1 |
| **II level SU** | 0 | 0 | 0 | 0 | 1 | 0 | 0 | 0 | 1 |
| **beSU** | 0 | 0 | 0 | 0 | 8 | 0 | 0 | 0 | 14 |
| **beTW** | 6 | 4 | 4 | 8 | 0 | ? | 8 | 0 | 64 |
| **MT24/7** | no* | no | no | no | yes | no | no | no | 1 |
| **N. of NIs** | 1 | 0 | 0 | 0 | 4 | 0 | 0 | 0 | 7 |

Legend: SU, stroke unit; beSU, beds available in SU; beTW, beds available in traditional wards; MT, Mechanical thrombectomy; NIs, Neuro interventionists;* the service is active, but not 24/7
