# Supplementary material for: Coverage of the requirements of first and second level stroke unit in Italy
Source: Neurol Sci. 2020 Jul 31;42(3):1073–9. doi: 10.1007/s10072-020-04616-x (PMC7870770; doi:10.1007/s10072-020-04616-x)
Supplement: Supplementary file 8 — (DOCX 20 kb) [file 10072_2020_4616_MOESM8_ESM.docx]

| **Region (4,375,865 inhab.)** | **Piemonte** | | | | | | | |
| --- | --- | --- | --- | --- | --- | --- | --- | --- |
| **Hospital/City** | Osp. Maria Vittoria-TORINO | Ospedale Martini - TORINO | ASLTO3 | Moncalieri ASLTO5 | ASL VCO | AOU Città della salute | AUO Maggiore della Carità-Novara | S. Giovanni Bosco-TORINO |
| **I level SU** | 1 | 0 | 1 | 1 | 1 | 0 | 0 | 0 |
| **II level SU** | 0 | 0 | 0 | 0 | 0 | 1 | 1 | 1 |
| **beSU** | 6 | 0 | 4 | 3 | 4 | 9 | 6 | 6 |
| **beTW** | 0 | 3 | 0 | 0 | 0 | 0 | 0 | 8 |
| **MT 24/7** | no | No | no | no | no | yes | yes | Yes |
| **N. of NIs** | 0 | 0 | 0 | 0 | 0 | 4 | 4 | 5 |

| **Region** | **Piemonte** | | | | | | |
| --- | --- | --- | --- | --- | --- | --- | --- |
| **Hospital/City** | S. Croce Carle-Cuneo | Ospedale San Lazzaro - Cuneo | ASLTO4 (Papurello) | ASLTO4 (Geda) | ASLCN1 PO Mondovi | ASL CN1 Osp. Savigliano | Pinerolo |
| **I level SU** | 0 | 0 | 0 | 0 | 0 | 0 | 1 |
| **II level SU** | 1 | 0 | 0 | 0 | 0 | 0 | 0 |
| **beSU** | 4 | 0 | 0 | 0 | 0 | 0 | 4 |
| **beTW** | 6 | 4 | **4** | 4 | 6 | 3 | 0 |
| **MT 24/7** | yes | No | no | no | no | no | No |
| **N. of NIs** | 5 | 0 | 0 | 0 | 0 | 0 | 0 |

| **Region** | **Piemonte** | | | | | | | **TOTAL** |
| --- | --- | --- | --- | --- | --- | --- | --- | --- |
| **Hospital/City** | ASL NO  Borgomanero  (Godi) | Ospedale degli Infermi Biella | Neurologia Novi/Tortona | Neurologia Casale Monferrato | AO ORDINE MAURIZIANO DI TORINO | AO SS Antonio e Biagio Alessandria | AOU SAN LUIGI |  |
| **I level SU** | 0 | 1 | 0 | 0 | 1 | 0 | 1 | 8 |
| **II level SU** | 0 | 0 | 0 | 0 | 0 | 1 | 0 | 5 |
| **beSU** | 0 | 4 | 0 | 0 | 6 | 4 | 6 | 66 |
| **beTW** | 4 | 0 | 6 | 2 | 0 | 0 | 0 | 50 |
| **MT 24/7** | no | no | no | no | no | no* | no | 4 |
| **N. of NIs** | 0 | 0 | 0 | 0 | 0 | 4 | 0 | 22 |

Legend: SU, stroke unit; beSU, beds available in SU; beTW, beds available in traditional wards; MT, Mechanical thrombectomy; NIs, Neuro interventionists;* the service is active, but not 24/7
