# Supplementary material for: Coverage of the requirements of first and second level stroke unit in Italy
Source: Neurol Sci. 2020 Jul 31;42(3):1073–9. doi: 10.1007/s10072-020-04616-x (PMC7870770; doi:10.1007/s10072-020-04616-x)
Supplement: Supplementary file 9 — (DOCX 18 kb) [file 10072_2020_4616_MOESM9_ESM.docx]

| **Region**  **(4.048.242 inhab )** | **Puglia** | **Puglia** | **Puglia** | **Puglia** | **Puglia** | **Puglia** | **Puglia** | **Puglia** | **Puglia** | **Puglia** | **Total** |
| --- | --- | --- | --- | --- | --- | --- | --- | --- | --- | --- | --- |
| **City or Town** | BARI  Policlinico | Taranto | BAT  (Barletta  Andria  Trani) | Lecce | Foggia | Brindisi | BARI  Di Venere | Altamura | Acquaviva (BA)  Miulli | S.G. Rotondo  (FG) |  |
| **I level SU** | 0 | 0 | 1 | 0 | 1 | 0 | 0 | 1 | 1 | 0 | 4 |
| **II level SU** | 1 | 1 | 0 | 1 | 0 | 1 | 0 | 0 | 0 | 1 | 5 |
| **beSU** | 12 | 6 | 6 | 6 | 6 | 6 | 0 | 4 | 6 | 4 | 56 |
| **beTW** | 28 | uk | 10 | uk | uk | uk | 6 | 8 | 20 | uk | 72 |
| **MT 24/7** | yes | yes | 0 | yes | 0 | No * | no | no | no | yes | 4 |
| **N. of NIs** | 3 | 3 | 0 | 4 | 0 | 2/3 | 0 | 0 | 0 | 4 | 16 |

Legend: SU, stroke unit; beSU, beds available in SU; beTW, beds available in traditional wards; MT, Mechanical thrombectomy ; NIs, Neuro interventionists ;* the service is active, but not 24/7; uk: unknown the number of available beds
