# Supplementary material for: Coverage of the requirements of first and second level stroke unit in Italy
Source: Neurol Sci. 2020 Jul 31;42(3):1073–9. doi: 10.1007/s10072-020-04616-x (PMC7870770; doi:10.1007/s10072-020-04616-x)
Supplement: Supplementary file 10 — (DOCX 27 kb) [file 10072_2020_4616_MOESM10_ESM.docx]

| **Region (1,531,735 inhab.)** | **Marche** | | | | | | | **Total** |
| --- | --- | --- | --- | --- | --- | --- | --- | --- |
| **City or Town** | Fermo | San Benedetto del Tronto | Ospedali Riuniti-Ancona | INRCA-Ancona | Jesi | Macerata | Osp. Riunuti Marche Nord Pesaro-Fano |  |
| **I level SU** | 1 | 1 | 0 | 1 | 1 | 1 | 1 | 6 |
| **II level SU** | 0 | 0 | 1 | 0 | 0 | 0 | 0 | 1 |
| **beSU** | 4 | 6 | 4 | 5 | 6 | 2 | 9 | 36 |
| **beTW** | 0 | 0 | 0 | 0 | 0 | 0 | 0 | 0 |
| **MT 24/7** | no | no | yes | no | no | no | no | 1 |
| **N. of NIs** | 0 | 0 | 4 | 0 | 0 | 0 | 0 | 4 |

Legend: SU, stroke unit; beSU, beds available in SU; beTW, beds available in traditional wards; MT, Mechanical thrombectomy; NIs, Neuro interventionists
