# Supplementary material for: Coverage of the requirements of first and second level stroke unit in Italy
Source: Neurol Sci. 2020 Jul 31;42(3):1073–9. doi: 10.1007/s10072-020-04616-x (PMC7870770; doi:10.1007/s10072-020-04616-x)
Supplement: Supplementary file 12 — (DOCX 24 kb) [file 10072_2020_4616_MOESM12_ESM.docx]

| **Region (**1,217,82 inhab.) | **Friuli-Venezia-Giulia** | | | | Total |
| --- | --- | --- | --- | --- | --- |
| **City/Town** | Udine | Pordenone | Trieste | Gorizia |  |
| **I level SU** | 0 | 1 | 0 | 1 | 2 |
| **II level SU** | 1 | 0 | 1 | 0 | 2 |
| **beSU** | 6 | 7 | 12 | 4 | 29 |
| **beTW** | 0 | 0 | 0 | 0 | 0 |
| **MT 24/7** | yes | no | yes | no | 2 |
| **N. of NIs** | 5 | 0 | 5 | 0 | 10 |

Legend: SU, stroke unit; beSU, beds available in SU; beTW, beds available in traditional wards; MT, Mechanical thrombectomy; NIs, Neuro interventionists
