# Supplementary material for: Coverage of the requirements of first and second level stroke unit in Italy
Source: Neurol Sci. 2020 Jul 31;42(3):1073–9. doi: 10.1007/s10072-020-04616-x (PMC7870770; doi:10.1007/s10072-020-04616-x)
Supplement: Supplementary file 13 — (DOCX 36 kb) [file 10072_2020_4616_MOESM13_ESM.docx]

| **Region (5,896,693 inhab.)** | **Lazio** | | | | | | | | |
| --- | --- | --- | --- | --- | --- | --- | --- | --- | --- |
| **Hospital/City** | Pertini ASL2-RO | Belcolle Viterbo | S. Eugenio-ASL2-ROMA | Policlinico Gemelli-ROMA | S. Filippo Neri ASL1-ROMA | Sant’Andrea ROMA | San Camillo Forlanini-ROMA | Tor Vergata-ROMA | Policlinico Umberto I (La Sapienza)-ROMA |
| **I level SU** | 0 | 0 | 1 | 0 | 0 | 0 | 0 | 0 | 0 |
| **II level SU** | 0 | 1 | 0 | 1 | 0 | 1 | 1 | 1 | 1 |
| **beSU** | 0 | 4 | 6 | 8 | 4 | 6 | 8 | 8 | 8 |
| **beTW** | 0 | 0 | 0 | 0 | 0 | 4 | 0 | 0 | 0 |
| **MT 24/7** | no | yes | no | yes | yes | yes | yes | yes | no* |
| **N. of NIs** | 0 | 4 | 0 | 5 | 3 | 5 | 6 | 7 | 3 |

| **Regione** | **Lazio** | **TOTAL** |
| --- | --- | --- |
| **Hospital/City** | S. Giovanni-Addolorata- ROMA |  |
| **I level SU** | 0 | 1 |
| **II level SU** | 0 | 7 |
| **beSU** | 0 | 52 |
| **beTW** | 4 | 8 |
| **MT 24/7** | yes | 7 |
| **N. of NIs** | 5 | 38 |

Legend: SU, stroke unit; beSU, beds available in SU; beTW, beds available in traditional wards; MT, Mechanical thrombectomy; NIs, Neuro interventionists ;* the service is active, but not 24/7
