# Supplementary material for: Coverage of the requirements of first and second level stroke unit in Italy
Source: Neurol Sci. 2020 Jul 31;42(3):1073–9. doi: 10.1007/s10072-020-04616-x (PMC7870770; doi:10.1007/s10072-020-04616-x)
Supplement: Supplementary file 14 — (DOCX 30 kb) [file 10072_2020_4616_MOESM14_ESM.docx]

| **Region**  **( 1,556,981 inhab.)** | **Liguria** | | | | | | | | **Total** |
| --- | --- | --- | --- | --- | --- | --- | --- | --- | --- |
| **City/town** | Galliera -Genova | San Martino-Genova | ASL4  Lavagna | ASL5 La Spezia | ASL3  Villa Scassi GE | ASL1 Imperia | ASL2 Savona | ASL2 Pietra Ligure |  |
| **I level SU** | 0 | 0 | 0 | 0 | 1 | 0 | 0 | 0 | 1 |
| **II level SU** | 0 | 1 | 0 | 0 | 0 | 0 | 0 | 1 | 2 |
| **beSU** | 0 | 12 | 0 | 0 | 8 | 0 | 0 | 4 | 24 |
| **beTW** | 4 | 2 | 1 | 8 | 0 | 6 | 1 | 0 | 22 |
| **MT 24/7** | no* | yes | no | no | no | no | no | yes | 2 |
| **N. of NIs** | 3 | 4 | 0 | 0 | 0 | 0 | 0 | 5 | 12 |

Legend: SU, stroke unit; beSU, beds available in SU; beTW, beds available in traditional wards; MT, Mechanical thrombectomy; NIs, Neuro interventionists;* the service is active, but not 24/7
