# Supplementary material for: Coverage of the requirements of first and second level stroke unit in Italy
Source: Neurol Sci. 2020 Jul 31;42(3):1073–9. doi: 10.1007/s10072-020-04616-x (PMC7870770; doi:10.1007/s10072-020-04616-x)
Supplement: Supplementary file 15 — (DOCX 53 kb) [file 10072_2020_4616_MOESM15_ESM.docx]

| **Region (**10,018,806 inhab.) | **Lombardia** | | | | | | | |
| --- | --- | --- | --- | --- | --- | --- | --- | --- |
| **Hospital/City** | Asst-valleolona.it | ASST Cremona | ASST Papa Giovanni XXIII, Bergamo | SARONNO | ASST OVEST MILANESE | asst-rhodense | s. carlo B | Crema |
| **I level SU** | 1 | 0 | 0 | 1 | 0 | 0 | 1 | 0 |
| **II level SU** | 0 | 1 | 1 | 0 | 0 | 0 | 0 | 0 |
| **beSU** | 6 | 0 | 6 | 6 | 0 | 0 | 8 | 0 |
| **beTW** | 0 | 8 | 0 | 0 | 8 | 7 | 0 | 6 |
| **MT 24/7** | no | yes | yes | no | no˟ | no | no˟ | no |
| **N. of NIs** | 0 | 1∞ | 1∞ | 0 | 3 | 0 | 3 | 0 |

| **Region** | **Lombardia** | | | | | | |
| --- | --- | --- | --- | --- | --- | --- | --- |
| **Hospital/City** | ASST Pavia | ASST Melegnano-Martesana | Istituto Clinico Città Studi | ospedale San Gerardo  Monza | Desio | ASST Lecco/Ospedale A. Manzoni | Sacco Fatebenefratelli, Milano |
| **I level SU** | 1 | 1 | 1 | 0 | 0 | 0 | 1 |
| **II level SU** | 0 | 0 | 0 | 1 | 1 | 1 | 0 |
| **beSU** | 4 | 10 | 8 | 12 | 14 | 4 | 4 |
| **beTW** | 0 | 0 | 0 | 0 | 0 | 0 | 0 |
| **MT 24/7** | no | no | no | yes | yes | yes | no |
| **N. of NIs** | 0 | 0 | 0 | 5 | 4 | 3 | 0 |

| **Region** | **Lombardia** | | | | | | |
| --- | --- | --- | --- | --- | --- | --- | --- |
| **Hospital/City** | Spedali Civili Brescia | Fondazione Poliambulanza Brescia | ASST Lecco - PO di Merate | Valduce | ASST lariana dip medico | Bassini | Poma Mantova |
| **I level SU** | 0 | 1 | 0 | 1 | 0 | 0 | 1 |
| **II level SU** | 1 | 0 | 0 | 0 | 1 | 0 | 0 |
| **beSU** | 10 | 4 | 0 | 6 | 8 | 0 | 15 |
| **beTW** | 0 | 0 | 6 | 0 | 0 | 0 | 0 |
| **MT 24/7** | yes | no ⃰ | no ⃰ | no ⃰ | yes | no | no ⃰ |
| **N. of NIs** | 5 | 2 | 1 | 2 | 5 | 0 | ? |

| **Region** | **Lombardia** | | | | | | | **Total** |
| --- | --- | --- | --- | --- | --- | --- | --- | --- |
| **Hospital/City** | San Matteo-Mondino | ASST SETTELAGHI | Fondazione IRCCS Ca' Granda Ospedale Maggiore Policlinico-MI | ASST Valtellina Alto Lario | ASST-Vimercate | ASST Grande Ospedale Metropolitano Niguarda-MI | Ist Auxologico It - H S.Luca |  |
| **I level SU** | 0 | 0 | 0 | 0 | 0 | 0 | 0 | 10 |
| **II level SU** | 1 | 0 | 1 | 0 | 0 | 1 | 1 | 11 |
| **beSU** | 12 | 0 | 6 | 0 | 0 | 14 | 4 | 161 |
| **beTW** | 0 | 6 | 0 | 4 | 6 | 0 | 0 | 57 |
| **MT 24/7** | yes | yes | yes | no | no | yes | yes | 12 |
| **N. of NIs** | 4 | 7 | 3 | 0 | 0 | 5 | 3 | 57 |

Legend: SU, stroke unit; beSU, beds available in SU; beTW, beds available in traditional wards; MT, Mechanical thrombectomy; NIs, Neuro interventionists * the service is active, but not 24/7; ∞ the service is active 24/7 thanks to other 3 operators in sharing with an other hub.
