# Supplementary material for: Coverage of the requirements of first and second level stroke unit in Italy
Source: Neurol Sci. 2020 Jul 31;42(3):1073–9. doi: 10.1007/s10072-020-04616-x (PMC7870770; doi:10.1007/s10072-020-04616-x)
Supplement: Supplementary file 16 — (DOCX 24 kb) [file 10072_2020_4616_MOESM16_ESM.docx]

| **Region (**4.448.841 inhab) | **Emilia-Romagna** | **Emilia-Romagna** | **Emilia-Romagna** | **Emilia-Romagna** | **Emilia**  **Romagna** | **Emilia-Romagna** | **Emilia-Romagna** |
| --- | --- | --- | --- | --- | --- | --- | --- |
| **City/Town** | Parma | Reggio E. | Modena | Ferrara | Ferrara II | Carpi | Ravenna |
| **I level SU** | 0 | 1 | 0 | 0 | 0 | 1 | 1 |
| **II level SU** | 1 | 0 | 1 | 1 | 0 | 0 | 0 |
| **beSU** | 8 | 12 | 16 | 18 | 0 | 4 | 13 |
| **beTW** | 0 | 0 | 0 | 0 | 3 | 0 | 0 |
| **MT 24/7** | yes | no* | yes | yes | no | no | no |
| **N. of NIs** | 3 | 3 | 8 | 5 | 0 | 0 | 0 |

Legend: SU, stroke unit; beSU, beds available in SU; beTW, beds available in traditional wards; MT, Mechanical thrombectomy ; NIs, Neuro interventionists ;* the service is active in daytime 2-3 days a week with a Drip&Drive model by Modena interventionists team.

| **Region** | **Emilia-Romagna** | **Emilia-Romagna** | **Emilia-Romagna** | **Emilia-Romagna** | **Emilia-Romagna** | **Emilia-Romagna** | **Emilia-Romagna** | **Total** |
| --- | --- | --- | --- | --- | --- | --- | --- | --- |
| **City/Town** | Bologna | Cesena | Piacenza | Forlì | Imola | Fidenza | Rimini |  |
| **I level SU** | 0 | 0 | 1 | 0 | 0 | 1 | 0 | 5 |
| **II level SU** | 1 | 1 | 0 | 0 | 0 | 0 | 0 | 5 |
| **beSU** | 20 | 6 | 12 | 0 | 0 | 6 | 0 | 115 |
| **beTW** | 0 | 0 | 0 | 6 | 4 | 0 | 4g | 17 |
| **MT 24/7** | yes | yes | no | no | no | no | no | 5 |
| **N. of NIs** | 6 | 5 | 0 | 0 | 0 | 0 | 0 | 30 |

Legend: SU, stroke unit; beSU, beds available in SU; beTW, beds available in traditional wards; MT, Mechanical thrombectomy ; NIs, Neuro interventionists ;* the service is active, but not 24/7
