# Supplementary material for: Coverage of the requirements of first and second level stroke unit in Italy
Source: Neurol Sci. 2020 Jul 31;42(3):1073–9. doi: 10.1007/s10072-020-04616-x (PMC7870770; doi:10.1007/s10072-020-04616-x)
Supplement: Supplementary file 17 — (DOCX 15 kb) [file 10072_2020_4616_MOESM17_ESM.docx]

| **Region (570.365 inhab)** | **Basilicata** | **Basilicata** | **Total** |
| --- | --- | --- | --- |
| **City/Town** | Matera | Potenza |  |
| **I level SU** | 1 | 1 | 2 |
| **II level SU** | 0 | 0 | 0 |
| **beSU** | 2 | 8 | 10 |
| **beTW** | 3 | 0 | 3 |
| **MT 24/7** | 0 | 0 | 0 |
| **N. of NIs** | 0 | 0 | 0 |

Legend: SU, stroke unit; beSU, beds available in SU; beTW, beds available in traditional wards; MT, Mechanical thrombectomy ; NIs, Neuro interventionists
