# Supplementary material for: Coverage of the requirements of first and second level stroke unit in Italy
Source: Neurol Sci. 2020 Jul 31;42(3):1073–9. doi: 10.1007/s10072-020-04616-x (PMC7870770; doi:10.1007/s10072-020-04616-x)
Supplement: Supplementary file 18 — (DOCX 25 kb) [file 10072_2020_4616_MOESM18_ESM.docx]

| **Region (1,956,687 inhab.)** | **Calabria** | | | | | **Total** |
| --- | --- | --- | --- | --- | --- | --- |
| **City/town** | Reggio Calabria | Vibo Valentia | Catanzaro | Crotone | Cosenza |  |
| **I level SU** | 0 | 0 | 1 | 0 | 0 | 1 |
| **II level SU** | 1 | 0 | 0 | 0 | 1 | 2 |
| **beSU** | 6 | 0 | 6 | 0 | 8 | 20 |
| **beTW** | 0 | 6 | 0 | 6 | 0 | 12 |
| **MT 24/7** | yes | no | no | no | yes | 2 |
| **N. of nNIs** | 7 | 0 | 0 | 0 | 9 | 16 |

Legend: SU, stroke unit; beSU, beds available in SU; beTW, beds available in traditional wards; MT, Mechanical thrombectomy; NIs, Neuro interventionists
