# Supplementary material for: Coverage of the requirements of first and second level stroke unit in Italy
Source: Neurol Sci. 2020 Jul 31;42(3):1073–9. doi: 10.1007/s10072-020-04616-x (PMC7870770; doi:10.1007/s10072-020-04616-x)
Supplement: Supplementary file 19 — (DOCX 37 kb) [file 10072_2020_4616_MOESM19_ESM.docx]

| **Region**  (5,839,084 inhab.) | **Campania** | | | | | | |
| --- | --- | --- | --- | --- | --- | --- | --- |
| **City/Town** | Cardarelli-Napoli | AO del Mare-Napoli | CTO dei Colli- Napoli | AOU San Giovanni e Ruggi-Salerno | AO Moscati-Avellino | AO San Pio - Benevento | AORN Caserta |
| **I level SU** | 0 | 0 | 0 | 0 | 0 | 1 | 0 |
| **II level SU** | 1 | 0 | 0 | 1 | 0 | 0 | 0 |
| **beSU** | 4 | 0 | 0 | 8 | 0 | 4 | 0 |
| **beTW** | 0 | 2 | 4 | 0 | 1 | 0 | 4 |
| **MT 24/7** | yes | no | no | yes | no˟ | no˟ | no |
| **N. of NIs** | 6 | 0 | 0 | 4 | 2 | 3 | 0 |

| **Region** | **Campania** | | | | **Total** |
| --- | --- | --- | --- | --- | --- |
| **City/Town** | FEDERICO II-Napoli | PO Nocera Inferiore -Salerno | Ariano Irpino-Avellino | Vallo della Lucania-Salerno |  |
| **I level SU** | 0 | 0 | 0 | 0 | 1 |
| **II level SU** | 1 | 0 | 0 | 0 | 3 |
| **beSU** | 2 | 0 | 0 | 0 | 18 |
| **beTW** | o | 1 | 4 | 3 | 19 |
| **MT 24/7** | yes | no˟ | no | no | 3 |
| **N. of NIs** | 5 | 2 | 0 | 0 | 22 |

Legend: SU, stroke unit; beSU, beds available in SU; beTW, beds available in traditional wards; MT, Mechanical thrombectomy; NIs, Neuro interventionists * the service is active, but not 24/7
