# Supplementary material for: Coverage of the requirements of first and second level stroke unit in Italy
Source: Neurol Sci. 2020 Jul 31;42(3):1073–9. doi: 10.1007/s10072-020-04616-x (PMC7870770; doi:10.1007/s10072-020-04616-x)
Supplement: Supplementary file 20 — (DOCX 28 kb) [file 10072_2020_4616_MOESM20_ESM.docx]

| **Region (1,315,196 inhab.)** | **Abruzzo** | | | | | | | |
| --- | --- | --- | --- | --- | --- | --- | --- | --- |
| **City/Town** | Avezzano | L’Aquila | Chieti | Pescara | Teramo | Lanciano | Vasto | Total |
| **I level SU** | 0 | 0 | 0 | 0 | 0 | 0 | 0 | 0 |
| **II level SU** | 0 | 0 | 0 | 1 | 0 | 0 | 0 | 1 |
| **beSU** | 0 | 0 | 0 | 17 | 0 | 0 | 0 | 17 |
| **beTW** | 6 | 4 | 4 | 0 | 6 | 6 | 3 | 29 |
| **MT 24/7** | no* | no* | no | yes | no* | no | no | 1 |
| **N. of NIs** | 2 | 3 | 0 | 4 | 2 | 0 | 0 | 11 |

Legend: SU, stroke unit; beSU, beds available in SU; beTW, beds available in traditional wards; MT, Mechanical thrombectomy; NIs, Neuro interventionists ;* the service is active, but not 24/7
